# Modulation of thalamic network connectivity using transcranial direct current stimulation based on resting-state functional magnetic resonance imaging to improve hypoxia-induced cognitive impairments

**DOI:** 10.3389/fnins.2022.955096

**Published:** 2022-08-25

**Authors:** Guo Dalong, Qin Yufei, Yang Lei, Li Pengfei, Ye Anqi, Guo Zichuan, Wang Cong, Zhou Yubin

**Affiliations:** Air Force Medical Center, Air Force Medical University, Beijing, China

**Keywords:** plateau hypoxia, transcranial direct current stimulation, functional connectivity, resting-state functional magnetic resonance imaging, cognitive function

## Abstract

Hypoxic conditions at high altitudes severely affect cognitive functions such as vigilance, attention, and memory and reduce cognitive ability. Hence, there is a critical need to investigate methods and associated mechanisms for improving the cognitive ability of workers at high altitudes. This study aimed to use transcranial direct current stimulation (tDCS) to modulate thalamic network functional connectivity to enhance cognitive ability. We recruited 20 healthy participants that underwent hypoxia exposure in a hypoxic chamber at atmospheric pressure to simulate a hypoxic environment at 4,000 m. Participants received both sham and real stimulation. tDCS significantly improved the participants’ emotional status, including depression, fatigue, and energy level. These effects were sustained for more than 6 h (*P* < 0.05 at the second to fifth measurements). In addition, tDCS enhanced vigilance, but this was only effective within 2 h (*P* < 0.05 at the second and third measurements). Central fatigue was significantly ameliorated, and cerebral blood oxygen saturation was increased within 4 h (*P* < 0.05 at the second, third, and fourth measurements). Furthermore, functional connectivity results using the thalamus as a seed revealed enhanced connectivity between the thalamus and hippocampus, cingulate gyrus, and amygdala after tDCS. These results indicated that tDCS increased local cerebral blood oxygen saturation and enhanced thalamic network connectivity in a hypoxic environment, thereby improving vigilance, depression, fatigue, and energy levels. These findings suggest that tDCS may partially rescue the cognitive decline caused by hypoxia within a short period. This approach affords a safe and effective cognitive enhancement method for all types of high-altitude workers with a large mental load.

## Introduction

The hypoxic environment at high altitudes affects the cardiovascular, digestive, and nervous system functions, thereby affecting cognitive functions, including learning, memory, and visual perception (Pun et al., 2018; Heinrich et al., 2019). This results in a cognitive decline in the inhabitants of high-altitude areas, especially those who rapidly enter high altitudes. Indeed, cognitive decline correlates with altitude, duration, and task complexity (Miguel and Silveira, 2020; Cai et al., 2021). In the past, high-altitude health protection programs predominantly included health education, nocturnal oxygenation, adjustment of dietary composition, and advancement of work and rest times (Srivastava and Kumar, 1992; Wing-Gaia, 2014; Tan et al., 2021). These methods focused on the protection of the respiratory and digestive systems. However, economic and technological developments, especially high-speed iteration of electronic equipment, have increased the complexity of work at high altitudes. This has led to a greater mental load in high-altitude workers, and traditional protection methods have had minimal effects on these types of work requiring a high cognitive load (Miguel and Silveira, 2020; Perosa et al., 2020; Wakhloo et al., 2020; Pang and Kim, 2021). Therefore, effective protection of cognitive function in high-altitude environments and improvements in mental work ability at high altitudes are urgent problems to be resolved.

Transcranial direct current stimulation (tDCS) is a neuromodulation technique. A weak direct current of 1–2 mA is applied between electrodes and placed on the scalp to stimulate target brain areas (Dalong et al., 2021). This non-invasive method alters the resting potential of neuronal membranes. tDCS is generally used to treat neurological diseases such as major depression, Parkinson’s disease, and chronic pain and to explore basic scientific questions regarding cognitive function (Kaminski et al., 2021). Recent studies have confirmed that this method can up-regulate or down-regulate the excitability of specific brain regions and improve cognitive ability in healthy individuals to perform specific tasks. Additionally, it enhances human performance in language, memory, mathematics, logic, attention, and coordination in the short term (Tzvi-Minker et al., 2020). tDCS is widely used to augment cognitive ability in view of these effects.

In recent years, tDCS has been reported to repair brain neural networks, thereby improving cognitive impairments caused by a decreased efficiency of functional connections in the brain. For example, tDCS improved attention and vigilance after sleep deprivation and reduced response bias during task performance (Nelson et al., 2014). In addition, we observed that anodal tDCS of the dorsolateral cortex enhanced connectivity between the thalamus-temporal cortex and thalamus-caudate nucleus, therefore, restoring the functional connectivity and global efficiency loss caused by fatigue and ameliorating cognitive impairments (Dalong et al., 2020). Due to its ability to enhance cognitive ability and ameliorate cognitive impairments, tDCS may restore hypoxia-induced decline in cognitive function, thus, providing a safe and effective method for cognitive enhancement in high-altitude workers with a large mental load. To the best of our knowledge, no study till date has evaluated the use of tDCS to improve hypoxia-induced cognitive decline. Furthermore, the exact mechanisms by which tDCS restores impaired cognitive function remain unknown.

Therefore, we hypothesized that hypoxia at high altitudes would decrease functional connectivity (FC) of the thalamic network, leading to cognitive impairments, and that tDCS would strengthen FC of the thalamic network, resulting in amelioration of cognitive impairment.

## Materials and methods

### Participants

The sample size was estimated using the G*Power program (v 3.1.9.7; Heinrich Heine University, Düsseldorf, Germany). When the effect size was set to 0.8, the significance level to 0.05, and the power to 0.95, the minimal total sample size was determined to be 16. A total of 20 healthy male participants without a history of neurological or psychiatric disease or medications were enrolled, assuming a 20% dropout rate. The mean age, body weight, and height were 21.2 ± 2.1 years, 63.4 ± 6.2 kg, and 173 ± 2.7 cm, respectively. The medical history of all the participants was assessed. Participants with a history of respiratory and nervous system diseases or those with a history of living at high altitudes and experience with high-altitude travel were excluded. Hypoxia screening was performed 1 week earlier. Participants were required to enter a hypoxic chamber for hypoxia screening for 1 h; those who experienced severe discomfort, severe headache, dizziness, and nausea were instructed to exit the chamber and were not re-tested. During the trial and within 1 week before the trial, they were instructed to avoid any drugs that affected neural excitability. In addition, participants were instructed to avoid coffee or tea during the trial, and all participants had regular work and rest times. This study complied with the Human Medical Research Ethics Guidelines, and the research protocol was approved by the Ethics Committee of the Air Force Medical Center according to the Code of Ethics of the World Medical Association (Declaration of Helsinki). All participants included in the trial completed an informed consent form.

### Transcranial direct current stimulation paradigm

**FIGURE 1:**
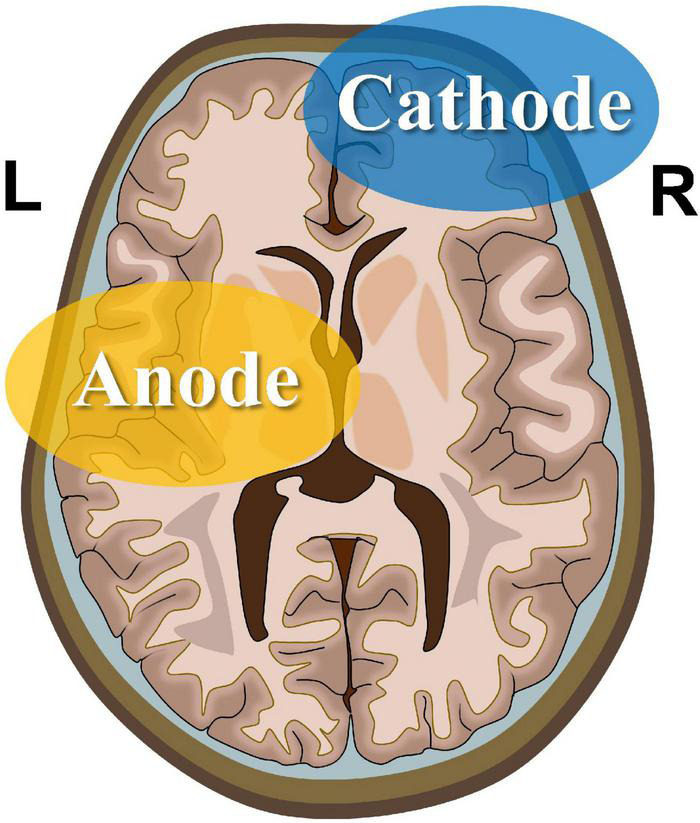
Transcranial direct current stimulation montage. Saline-soaked anodal sponge electrodes (5 cm × 5 cm) are applied over the left M1 region, and a larger cathodal electrode (5 cm × 7 cm) is placed on the right supraorbital area.

The experimental procedure involved a tDCS device certified by CE (NeuroConn, Ilmenau, Germany). The electrode configuration is presented in Figure 1. First, a saline-soaked sponge electrode (5 cm × 5 cm) was applied to the left M1 area with a medical bandage as the anode. Subsequently, a larger cathode electrode (5 cm × 7 cm) was positioned on the right supraorbital area. This large-area electrode method reduced the risk of cauterization by the current and reduced skin irritation (Mckinley et al., 2013; McIntire et al., 2014; Vancleef et al., 2016). During the real stimulation, the constant current was raised to 2 mA within 10 s for 20 min. To ensure safety, the maximum current density was always ≤0.08 mA/cm^2^ (Bikson et al., 2009). During the sham stimulation, the same electrode configuration was used, except that the current only lasted for 30 s, after which the current intensity was reduced to zero to induce itching without any significant effect on the cerebral cortex. Previous fMRI studies with tDCS treatment have demonstrated that this sham stimulation only exerted slight effects on the cerebellum without a significant effect on brain functional connectivity (Park et al., 2013).

### Testing indicators and environment

The profile of mood states (POMS) is one of the most commonly used scales to measure emotional states. The POMS contains six subscales: tension-anxiety (TA), depression-dejection (DD), anger-hostility (AH), fatigue-inertia (FI), confusion-bewilderment (CB), and vigor-activity (VA) (Morfeld et al., 2007). The scale comprises 65 items, each of which is divided into five options with corresponding scores as follows: 0 (insignificant); 1 (mild); 2 (moderate); 3 (equivalent); and 4 (extreme). For the first five subscales, higher scores indicate worse mood (negative scale); the opposite is true for VA scores (positive scale). The six subscales have high internal consistency and test-retest reliability. This simple and efficient method enables the simultaneous measurement of six emotional states (Morfeld et al., 2007).

The psychomotor vigilance task (PVT) detects sustained attention to visual target stimuli. It has the advantages of simple operation, fast experimental data processing, small learning effects, and high detection sensitivity (Reifman et al., 2018). Participants were required to click on the screen when the target visual stimulus appeared, and it was presented randomly and quickly. Advance operations, incorrect pressing, and excessive clicking time were regarded as errors and automatically recorded. In this study, CANTAB (Cambridge Cognition, Cambridge, United Kingdom) was used to measure attentional changes based on measurements of reaction times.

The critical flicker frequency (CFF) refers to the absolute threshold for measuring the visual response to light, which permits measurement and evaluation of central nervous system fatigue levels and objectively reflects mental workload. During testing, participants were required to regulate the portable HEPAtonorm Analyzer (nevoLAB GmbH, Maierhöfen, Germany) to adjust the flicker frequency of a bright red spot. The analyzer started at a frequency of 60 Hz, which appeared to the subject as a blink-free spot, and then decreased gradually. When the subject noticed that the red spot started flickering, he pressed the controller button of the analyzer, and the CFF was recorded (Feshchenko et al., 1994; Maier et al., 2017).

Regional oxygen saturation (*rSO*_2_) reflects changes in the homeostatic relationship between cerebral oxygen supply and demand. This parameter enables direct and continuous monitoring of cerebral hypoxia in a non-invasive manner. Compared to the measurement of arterial oxygen saturation at the fingernail, *rSO*_2_ accurately reflects the state of cerebral hypoxia and changes in cognitive function (Wang et al., 2019). We used a wireless cerebral blood oxygen acquisition system (Worth, Casibrain Technology, China); the participants were fitted with the signal acquisition headband. They could connect to the testing tablet via Bluetooth to enable real-time recording of cerebral blood oxygen saturation changes.

After the test had commenced, the PVT and POMS of each participant were tested, followed by the CFF test. The local cerebral blood oxygen saturation of the participants was collected simultaneously. The test was performed in the atmospheric hypoxic chamber of the Air Force Medical Center. The facility reduced oxygen concentration in the confined space by filling it with nitrogen, therefore, simulating the hypoxic environment at high altitudes. In principle, the partial pressure of oxygen in the hypoxia chamber was consistent with the partial pressure of oxygen at the simulated altitude. This method is simple, low-cost, and has good repeatability which also could avoid decompression sickness (Loeppky et al., 1997; Richard and Koehle, 2012; Mirna et al., 2020). Some studies have indicated that for short but intense hypoxic exposures, this normobaric hypoxia method could serve as an alternative of similar effect to hypobaric hypoxia (Richard and Koehle, 2012; Tao et al., 2014; Guo et al., 2015; Chan et al., 2021; Lauterbach et al., 2021).

### fMRI protocol and image analysis

Resting-state fMRI (rsfMRI) was performed at the Air Force Medical Center (Beijing, China) using a SIEMENS Magnetom Trio Tim 3.0T equipped with a 12-channel head matrix coil. Three scans (baseline, post-sham, and post-tDCS) were performed for each participant in the scanner suite prior to the sham tDCS and after the real and sham tDCS.

At the initial stage of the baseline scan, a high-resolution T1-weighted anatomical image was acquired using MPRAGE sequence [echo time (TE) = 9 ms, flip angle = 150°, repetition time (TR) = 2,000 ms, field of view (FOV) = 320 mm × 320 mm, 196 slices, voxel size = 1 mm× 1 mm× 1 mm). Afterward, three consistent functional scans were implemented employing standard echo-planar imaging sequence. Scans consisted of 210 functional images (TE = 30 ms, flip angle = 90°, TR = 2 s, FOV = 256 mm× 256 mm, 45 slices, voxel size = 3 mm× 3 mm× 4 mm, matrix size = 64 × 64, gap = 1 mm). During the scans, participants were required to remain relaxed, breathe steadily, stay sober, and fixate on a visual cross target.

The rsfMRI data were preprocessed using SPM12^1^ in MATLAB (TheMathWorksInc., Natick, MA, United States). The first five volumes were eliminated, followed by time correction, realignment, regression, filtering (0.01–0.08 Hz), normalization, and smoothing. Then, the CONN toolbox was adopted to perform seed-to-whole-brain FC analyses. The thalamus, which acts as an information relay between different subcortical areas and the cerebral cortex, was selected as the seed to explore its role in cognitive rehabilitation (Ionta et al., 2014).

The first FC analysis was conducted, and a time series correlation analysis was performed between the time series of the thalamus and all brain voxels to produce seed-to-whole-brain Fisher transformation maps. Subsequently, group-level analyses were conducted utilizing these maps in CONN. Repeated-measures ANOVAs with baseline, post-sham, and post-tDCS FC maps were used to calculate the main effects. The contrasts were set to baseline > post-sham and post-tDCS > post-sham with an aim to study the hypoxia and tDCS caused effects, respectively, and the analysis was controlled according to age. The cluster-level threshold was *P* < 0.05, and the false discovery rate was corrected, with >100 voxels per cluster.

To examine the relationship between changes in FC after anodal stimulation and cognitive function under the real tDCS condition, we performed a linear regression analysis of each participant’s FC difference (real—sham) with reaction times of PVT, CFF, and *rSO*_2_ as the regressors of interest. The statistical significance threshold was set at *P* < 0.05, Bonferroni-corrected for multiple comparisons.

### Experimental procedures

**FIGURE 2:**
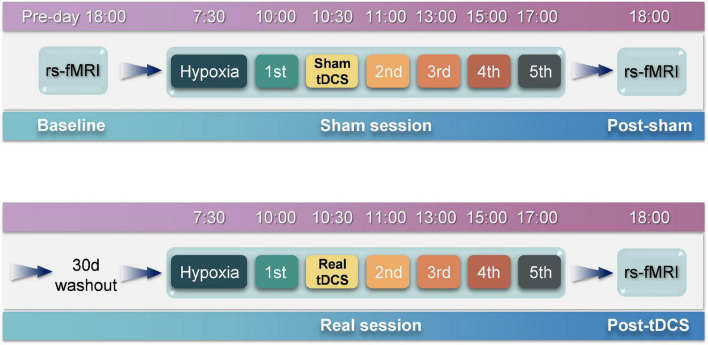
Experimental procedures. The entire experiment consists of two sessions, i.e., sham and real sessions. The real session is separated from the sham session by 30 days to eliminate after-effects. rsfMRI scans are performed before and after the sham session to obtain baseline and post-sham functional images. After the real session, another rsfMRI scan is performed to obtain post-tDCS functional images.

rsfMRI scans were performed at 18:00 h on the day before the experiment (Figure 2). On the day of the experiment, participants entered the atmospheric pressure hypoxia chamber at 07:30 h. The chamber was filled with nitrogen to reduce the oxygen concentration, such that the partial pressure of oxygen simulated an altitude of 4,000 m (the partial pressure of oxygen at this time was 12.63 kPa), during which participants were not informed of the simulated altitude. The participants were allowed to perform activities with minimal physical exertion, such as watching movies or reading books, and vigorous exercise was not permitted. The first test was performed 2.5 h later. On completion of the test, the physician passed the tDCS device with pre-set parameters to the experimenter for implementation. The study adopted a double-blind design, as neither the participants nor the experimenter was aware of whether the stimulus was real or sham. After the stimulation was completed, five tests were conducted at 11:00, 13:00, 15:00, and 17:00 h, respectively. A rsfMRI scan was performed at 18:00 h. At least one month after the wash-out period, the above process was repeated, except that the tDCS segment was replaced by the real stimulation program.

The experiment was conducted as depicted in Figure 2. Five participants were selected for the experiment using a random-walk sampling method. Throughout the experiment, participants stayed in the hypoxia chamber with the exception of toilet breaks and rsfMRI scans to ensure that continuous hypoxia was maintained. During the experiment, participants were continually observed; those who exhibited severe reactions to hypoxia were instructed to exit the hypoxia chamber immediately.

Data were processed with SPSS software (v 18.0, Armonk, NY, United States). The results are expressed as $\bar{X}$ *S*. The One-Sample Ryan-Joiner test based on the Shapiro-Wilk correction was used to determine if the data were normally distributed. Repeated-measures analysis of variance (RM-ANOVA) was used to evaluate the effect of tDCS, and Mauchly’s test of sphericity was used to inspect whether the sample met the assumptions of the RM-ANOVA. The degrees of freedom were corrected if the conditions were not satisfied, and *P* < 0.05 was considered statistically significant. Multivariate ANOVA with the Bonferroni correction was used to analyze the two groups of data with the same measurements, and *P* < 0.01 was considered statistically significant.

## Results

The performance of all participants conformed to the criteria above. None of the participants reported symptoms of skin burns, persistent vertigo, or nausea.

### Profile of mood states

**FIGURE 3:**
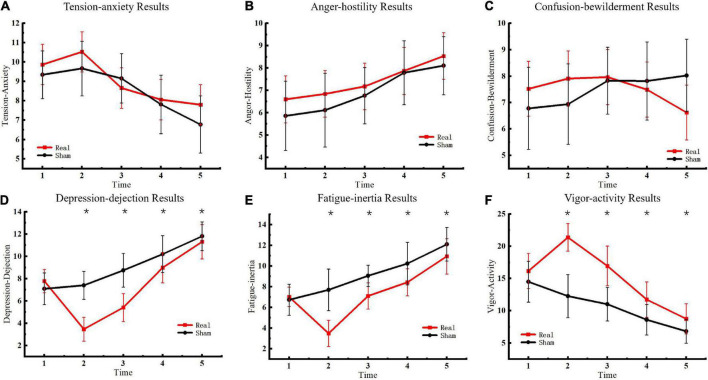
Profile of mood states results. **(A)** Tension-anxiety results. **(B)** Anger-hostility results. **(C)** Confusion-bewilderment results. **(D)** Depression-dejection results. **(E)** Fatigue-inertia results. **(F)** Vigor-activity results. Asterisks indicate significant differences between real and sham tDCS conditions.

The emotional state of participants was measured five times before and after sham or real tDCS, respectively, and the results of the POMS scale were classified (Figure 3). No significant differences were observed in TA, AH, and CB scales between the experimental and control groups. In contrast, significant differences were observed in DD [*F*_(4, 76)_ = 1163.52, *P* < 0.01], FI [*F*_(4, 76)_ = 220.62, *P* < 0.01], and VA [*F*_(4, 76)_ = 406.20, *P* < 0.01] scales between the experimental and control groups. Furthermore, for DD, significant between-group differences were observed at the second [*F*_(1, 19)_ = 137.05, *P* < 0.01], third [*F*_(1, 19)_ = 69.19, *P* < 0.01], fourth [*F*_(1, 19)_ = 15.00, *P* < 0.01], and fifth [*F*_(1, 19)_ = 9.00, *P* < 0.01] measurements. For FI, significant between-group differences were observed at the second [*F*_(1, 19)_ = 74.43, *P* < 0.01], third [*F*_(1, 19)_ = 34.27, *P* < 0.01], fourth [*F*_(1, 19)_ = 13.05, *P* < 0.01], and fifth [*F*_(1, 19)_ = 12.65, *P* < 0.01] measurements. For VA, significant between-group differences were observed at the second [*F*_(1, 19)_ = 124.08, *P* < 0.01], third [*F*_(1, 19)_ = 51.81, *P* < 0.01], fourth [*F*_(1, 19)_ = 17.92, *P* < 0.01], and fifth [*F*_(1, 19)_ = 9.99, *P* < 0.01] measurements. Although the difference between the two groups decreased over time, the results of the two groups remained significantly different at the fifth measurement.

### Psychomotor vigilance task

**FIGURE 4:**
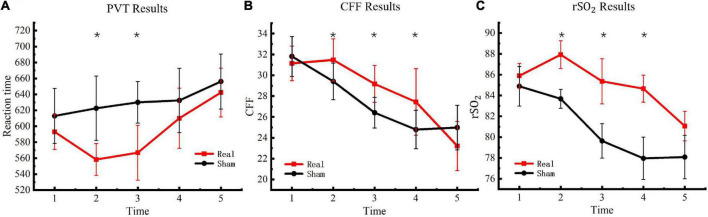
Critical flicker frequency and regional oxygen saturation results. **(A)** PVT results. **(B)** CFF results. **(C)**rSO_2_ results. Asterisks indicate significant differences between real and sham tDCS conditions.

The PVT task was used to measure participants’ psychomotor vigilance five consecutive times. Reaction times in the PVT task were used to determine changes in vigilance after entering the hypoxic environment. The results are presented in Figure 4A. Significant differences were observed between the experimental and control groups [*F*_(4, 76)_ = 4.56, *P* < 0.01] at the second [*F*_(1, 19)_ = 37.41, *P* < 0.01] and third [*F*_(1, 19)_ = 37.40, *P* < 0.01] measurements but not at the fourth [*F*_(1, 19)_ = 3.39, *P* = 0.08] or fifth measurements [*F*_(1, 19)_ = 6.32, *P* = 0.02].

### Critical flicker frequency

To evaluate changes in the degree of central nervous system fatigue of the participants in a hypoxic environment, CFF was detected with a digital bright spot scintillator five consecutive times. The final test results are presented in Figure 4B. Significant differences were observed between the experimental and control groups [*F*_(4, 76)_ = 10.77, *P* < 0.01] at the second [*F*_(1, 19)_ = 11.75, *P* < 0.01], third [*F*_(1, 19)_ = 23.58, *P* < 0.01], and fourth measurements [*F*_(1, 19)_ = 9.59, *P* < 0.01] but not at the fifth measurement [*F*_(1, 19)_ = 3.92, *P* = 0.06].

### Regional oxygen saturation

The WORTH headband was used to measure participants’ cerebral blood regional oxygen saturation five consecutive times to evaluate changes in cerebral blood oxygen saturation of the participants under a hypoxic environment. The test results are presented in Figure 4C. Significant differences were observed between the experimental and control groups [*F*_(4, 76)_ = 16.39, *P* < 0.01] at the second [*F*_(1, 19)_ = 108.30, *P* < 0.01], third [*F*_(1, 19)_ = 71.16, *P* < 0.01], and fourth measurements [*F*_(1, 19)_ = 149.31, *P* < 0.01] but not at the fifth measurement [*F*_(1, 19)_ = 2.44, *P* = 0.13].

### Functional connectivity changes with sham and real stimulation

**FIGURE 5:**
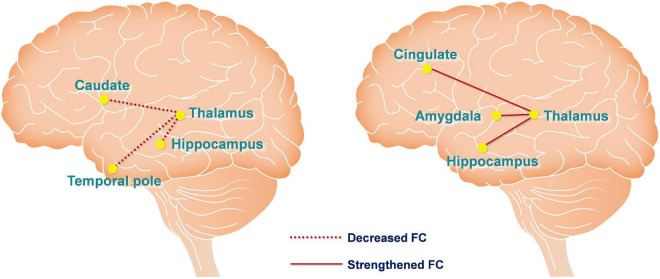
Functional connectivity changes are caused by hypoxia and anodal tDCS. The left figure depicts hypoxia-induced FC changes of the thalamic network using the contrast of baseline > post-sham. The right figure depicts anodal tDCS-induced FC changes of the thalamic network in the hypoxia environment using the contrast of post-tDCS > post-sham. The red dashed and solid lines represent decreased and increased FC, respectively.

We measured FC changes comparing baseline > post-sham and post-tDCS > post-sham using the thalamus as a seed (Table 1). After hypoxic exposure (baseline > post-sham), FC between the thalamus seed and right temporal pole, hippocampus, and left caudate was decreased, whereas after anodal stimulation (post-tDCS > post-sham), FC between the thalamus seed and cingulate gyrus, amygdala, and hippocampus was increased (Figure 5).

**TABLE 1:** Main effects of hypoxic environment and tDCS on FC of the thalamic network.

Region (label)	T-statistics	Cluster size	Cluster *P*-value
**Baseline > post-sham**			
TP l	0.23	1029	<0.001
Hippocampus	0.52	586	<0.01
TP r	0.18	426	<0.01
Caudate l	0.31	325	<0.01
**Post-tDCS > post-sham**			
Cingulate gyrus	5.21	568	<0.001
Amygdala	3.17	456	<0.01
Hippocampus	0.59	329	<0.01

The main effects of anodal tDCS on FC comparing baseline > post-sham and post-tDCS > post-sham using the thalamus as a seed. T-statistics and cluster P-values correspond to the peak voxels within the anatomical region(s) specified in the left column. For all contrasts, increased activity is reported at a cluster level threshold of P < 0.05 (false discovery rate-corrected). FC, functional connectivity; tDCS, transcranial direct current stimulation; TP l, left temporal pole; TP r, right temporal pole.

**FIGURE 6:**
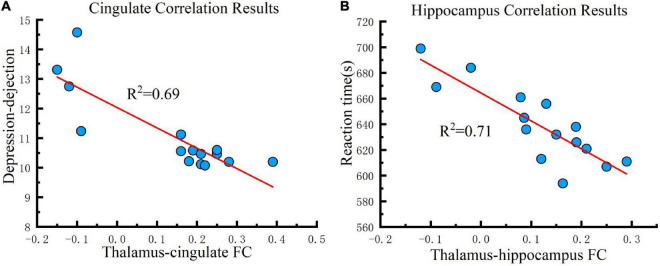
Correlation results. **(A)** Cingulate correlation results. FC changes between the thalamus and cingulate are correlated with depression-dejection in the fifth test across all participants under the real tDCS condition. **(B)** Hippocampus correlation results. FC changes between the thalamus and hippocampus are correlated with the reaction time of PVT in the fifth test across all participants under the real tDCS condition. The coefficient of determination denoted *R*^2^ in the figure indicates the proportion of depression-dejection that could be predicted from FC variation. FC, functional connectivity.

For investigating the influence of the strengthened FC that the tDCS caused to cognition, correlation analysis was conducted between FC changes (post-tDCS > post-sham) and POMS, PVT, CFF, and *rSO*_2_ under the real tDCS condition for the fifth test. The change in FC between the thalamus seed and cingulate was negatively correlated with the values for DD in POMS under the real condition (*P* < 0.001) (Figure 6A). In contrast, an increase in FC between the seed and hippocampus was negatively correlated with reaction time in the PVT under the real tDCS condition (*P* < 0.001) (Figure 6B).

## Discussion

Transcranial direct current stimulation has been proven to enhance cognitive abilities for performing specific tasks, including language, memory, mathematics, logic, attention, and coordination. Indeed, the United States Air Force has proposed the usefulness of this technology in military warfare for brain control (Nelson et al., 2014). However, there is a paucity of research on tDCS technology to improve cognitive function in hypoxic environments. This study explores whether anodal tDCS could improve cognitive function under hypoxia, improving the brain’s ability to function in a high-altitude environment. Distinct from previous studies, this study was conducted in a hypoxic environment that simulated a plateau, through which the brain was exposed to hypoxia, leading to a decline in cognition and brain function. In this setting, we used tDCS to stimulate the motor cortex of the human brain. This study employed psychological scales alongside objective indicators such as cerebral blood oxygen saturation, CFF, and rsfMRI to comprehensively evaluate the impact of tDCS technology on cognitive function after hypoxia.

This analysis revealed that tDCS improved emotional state. Compared to the sham stimulation group, participants’ depressive state was reduced, fatigue was ameliorated, and energy levels were increased following tDCS. These effects persisted until the fifth measurement. In this regard, the effects of tDCS on mood could be sustained for up to 6 h (11:00–17:00 h). However, there were no significant differences in the TA, AH, and CB scales. Further, tDCS did not significantly affect tension, anger, and confusion. These results indicated that tDCS had a more significant impact on certain emotions, which could be related to the electrical stimulation of specific brain areas. This study targeted the motor cortex involved in planning, control, and exercise. Studies have confirmed that electrical stimulation of this area improves energy and vigilance and alleviates symptoms of depression (Fregni et al., 2005; Douglas et al., 2015). The results of this study were consistent with previous findings. The test results demonstrated that stimulation of the primary motor cortex with the tDCS anode improved depression and fatigue and enhanced energy (Gálvez et al., 2013; Martin et al., 2014). In summary, anodal tDCS on the primary motor cortex ameliorated the hypoxia-induced depressive and fatigue states and improved energy levels, all of which are critical factors for plateau working.

Response times in the PVT task increased gradually in the hypoxic environment for both the experimental and control groups. This suggested that hypoxic conditions resulted in reduced responsivity, sustained attention, and vigilance of the human brain. tDCS significantly decreased participants’ response time and increased response speed, but these effects were not observed in the control group. These findings indicated that tDCS anodal stimulation enhanced sustained attention. However, this effect only lasted for 2 h, and there was no significant difference between the fourth and fifth measurements. These results are consistent with the findings of the United States Air Force Research Laboratory, which demonstrated that tDCS improved vigilance and sustained attention of aircraft dispatchers under simulation (Nelson et al., 2014). In short, anodal tDCS on the primary motor cortex counteracted the loss of attention caused by the hypoxic environment, and this effect was evident for 2 h.

The CFF is a key indicator of the mental load of workers and enables an effective evaluation of central nervous system fatigue. These results demonstrated that the CFF of the experimental and control groups decreased significantly over time, and the hypoxic environment reduced the CFF. At the second measurement, CFF was significantly higher in the real group than in the sham group, indicating that tDCS delayed brain fatigue. However, this difference only lasted until the fourth measurement, suggesting that the beneficial effect of tDCS on brain fatigue lasted for 4 h. Notably, there was no significant difference between the groups at the fifth measurement, but the reaction time of the real group was longer, implying that tDCS-induced improvements in fatigue may deplete energy reserves, a hypothesis that needs to be further studied. Indeed, tDCS may delay the process of fatigue, rather than allow the brain to rest.

The results of cerebral blood regional oxygen saturation revealed that the cerebral regional blood oxygen level decreased significantly with exposure to a hypoxic environment. The results of the real stimulation group demonstrated that tDCS anodal stimulation increased localized cerebral blood oxygen levels, and this effect was maintained for 4 h. The United States Air Force Research Laboratory reported that tDCS increased cerebral regional oxygen saturation in fatigued individuals, and transcranial Doppler demonstrated that tDCS increased cerebral blood flow velocity (Nelson et al., 2014). The results of this study revealed that tDCS increased localized cerebral blood oxygen saturation under hypoxic conditions, thereby improving cerebral hypoxia. The data obtained from this study provide a physiological basis for harnessing this technology to mitigate high-altitude cerebral hypoxia.

The current results demonstrated that tDCS anodal stimulation of the motor cortex improved the emotional status of participants, especially depression and energy status. Of note, these effects were sustained for more than 6 h. Nevertheless, the technology did not improve tension, anger, or confusion. In addition, this technology acutely relieved brain fatigue and improved vigilance, but this was only effective for 2 h in this study. We observed that tDCS did not consistently improve the duration of vigilance and mood in hypoxic environments. tDCS resulted in more sustained improvements in mood and a shorter increase in vigilance. This indicates that in a hypoxic environment, the effects of a single tDCS will last for more than 6 h if the goal is to improve mood. However, if the intention is to maintain vigilance and relieve brain fatigue for an extended period of time during high-intensity continuous work, multiple stimulations should be administered at intervals of more than 2 h or the stimulation intensity should be increased. Previous studies have suggested that multiple stimulations significantly prolong stimulation effects, and stronger current strength also exerted sustained effects (Bikson et al., 2009).

Regarding the rsfMRI results, we applied seed-based connectivity analysis to study the FC changes of the thalamus network caused by hypoxia and tDCS using a preset contrast: baseline > post-sham and post-tDCS > post-sham. The hippocampus is located between the thalamus and medial temporal lobe and is a component of the limbic system, which is generally considered to be associated with memory storage, switching, and orientation. The hippocampus converts short-term memories into long-term memories by consolidating the former through long-term potentiation (Goldfarb et al., 2016; Kim et al., 2017). The results of this study demonstrated that hypoxia reduced FC between the thalamus and hippocampus, and tDCS reversed this change, such that FC between the thalamus and hippocampus was rescued and enhanced. In addition, FC of the thalamus, cingulate gyrus, and amygdala was also enhanced after tDCS. It is generally believed that the anatomical basis of emotion comprises circuitry between the mammillary body, anterior nucleus of the thalamus, cingulate gyrus, hippocampus, and amygdala. Accordingly, the current findings suggest that tDCS may improve emotional state under hypoxia by enhancing FC of the thalamus with the cingulate and amygdala.

The magnitude of the connectivity enhancement between the thalamus and cingulate was negatively correlated with the value of DD at the fifth measurement, suggesting that tDCS could improve depression and dejection by enhancing FC between the thalamus and cingulate. The cingulate gyrus regulates emotions via thalamocortical circuits. Depression and other mental disorders may manifest when activation of this brain region is decreased for a prolonged period (Whalley et al., 2009). Statistically, individuals with a family history of depression are more likely to have abnormal functional connections between the thalamus and cingulate gyrus, which may lead to dysfunction in emotional regulation (Whalley et al., 2015). The results of this study also support the conclusion that connections between the thalamus and cingulate gyrus are strongly correlated with depression. The use of tDCS to enhance connectivity between the thalamus and cingulate gyrus may improve emotional regulation, consequently, ameliorating symptoms of depression and dejection caused by hypoxia.

Also, the magnitude of the connectivity enhancement between the thalamus and hippocampus was negatively correlated with the value of reaction time at the fifth measurement, suggesting that tDCS improved attention by enhancing FC between the thalamus and hippocampus. In addition to its well-established role in memory, the hippocampus has recently been implicated in guiding attention. Memory of complex scenes engages the hippocampus and facilitates attention and eye movements to targets, even in the absence of explicit recall (Goldfarb et al., 2016). The results show that anodal tDCS reduced reaction times by enhancing FC in the thalamus and hippocampus, implying that the connectivity between the thalamus and hippocampus was strongly correlated with attention. Given that the FC between the thalamus and hippocampus was reduced under hypoxic conditions, the data suggest that hypoxia impairs memory and attention by reducing connectivity between the thalamus and hippocampus, and tDCS may rescue these defects induced by hypoxia, ultimately improving memory and attention.

The thalamus has strong reciprocal connections with the cerebral cortex, forming a network that is believed to be involved with many cognitive functions (Ehud and Tess, 2015). The results show that the thalamus is well-placed to regulate the interactions between distributed brain regions that perform different cognitive functions in the cerebral cortex. When the brain is exposed to a hypoxic environment, the connection between the thalamus and cortical regions is impaired and then cognitive function corresponding to the brain region decreases. Conversely, when anodal tDCS improves the connection of the thalamocortical network, cognitive function will recover accordingly. Our previous research also supports this theory; the connectivity of the thalamus with the postcentral gyrus and temporal pole decreased after sleep deprivation, which resulted in lapses in alertness, and anodal tDCS improved vigilance by strengthening the connection between the thalamus and cortex (Dalong et al., 2020). This theory also suggests that tDCS could be a useful tool to repair the connection of the thalamocortical network and in turn regain motivated cognitive competence. In fact, many previous studies support these findings. For example, Leonie Steiner et al., using rsfMRI, found that connectivity strength of thalamocortical networks was associated with cognitive abilities, including but not limited to the speed of information processing, attention, and cognitive flexibility. Furthermore, they also found better cognitive abilities were correlated with a strengthened connection of the thalamocortical network in the mediodorsal nucleus and nuclei from the lateral group (Steiner et al., 2020). In short, the thalamus network could be the gate to improving cognitive functions, and anodal tDCS could be the key to opening it.

Despite these novel findings, this study has several limitations. The first limitation of this study is the number of subjects included, which undoubtedly reduced the statistical power and increased the likelihood of false positives. Second, the study should analyze how long the effect of hypoxia on the brain can last and should recruit different participants for the experimental and control groups. Third, we first performed sham tDCS and then the real one for all the participants, which could cause some error, since the order of sham and real stimulation should be counterbalanced for both groups. Furthermore, although all participants had undergone preliminary examinations and agreed to avoid psychotropic drugs and other psychostimulant beverages for 1-week before the trial, the lack of further clinical examinations limited the rigor of this trial. Therefore, future studies with larger sample sizes and more rigorous trials are warranted to validate our conclusions.

## Conclusion

The results of this study demonstrated that anodal tDCS on the left M1 induced immediate and extended effects that suppressed the FC of the thalamus with the cingulate, amygdala, and hippocampus. These effects in turn ameliorated hypoxia-induced depression and fatigue, and improved vigor, attention, and rSO_2_. Also, the results demonstrated that the thalamic network plays a key role in the regulation of mood states and cognition. The hypoxic environment disrupted the connectivity of the thalamus with the caudate, hippocampus, and temporal pole while anodal tDCS strengthened the network between the thalamus and cingulate, amygdala, and hippocampus, which in turn improved mood and enhanced cognitive performance. As tDCS of the left M1 modulated the thalamic network and resulted in the aforementioned beneficial effects, this technique may improve operational capacity in high-altitude areas without an additional oxygen supply. In conclusion, tDCS affords a safe and effective method of cognitive function enhancement for high-altitude workers and residents with high mental load.

## Data availability statement

The raw data supporting the conclusions of this article will be made available by the authors, without undue reservation.

## Ethics statement

The studies involving human participants were reviewed and approved by the Ethics Committee of the Air Force Medical Center. The patients/participants provided their written informed consent to participate in this study.

## Author contributions

GD: conception and study design. GD, GZ, and ZY: data collection or acquisition. ZY, WC, and QY: statistical analysis. GD and ZY: interpretation of results. GD, YA, QY, ZY, and WC: drafting the manuscript or revising it critically for important intellectual content. All authors approved the final version to be published and agreement to be accountable for the integrity and accuracy of all aspects of the work.

## Footnotes

1
http://www.fil.ion.ucl.ac.uk/spm/software/spm12/

## Abbreviations

CFF: critical flicker frequency
FC: functional connectivity (FC) of the thalamic network, leading to cognitive impairments
fMRI: resting state magnetic resonance imaging
POMS: profile of mood states
tension-anxiety: TA
depression-dejection: DD
anger-hostility: AH
fatigue-inertia: FI
confusion-bewilderment: CB
vigor-activity: VA
PVT: psychomotor vigilance task
rSO_2_: regional oxygen saturation
tDCS: transcranial direct current stimulation
TE: echo time
TR: repetition time
FOV: field of view.
